# Supplementary material for: An open-source, lockable mouse wheel for the accessible implementation of time- and distance-limited elective exercise
Source: PLoS One. 2021 Dec 21;16(12):e0261618. doi: 10.1371/journal.pone.0261618 (PMC8691618; doi:10.1371/journal.pone.0261618)
Supplement: Attachment — Submitted filename: response to reviewers.docx [file pone.0261618.s003.docx]

**Dear Dr. Smeyne,**

**We are excited to resubmit our manuscript, “An open-source, lockable mouse wheel for the accessible implementation of time- and distance-limited elective exercise” to PLOS One. The comments we received were helpful to complete the revision and are addressed below.**

**Best Regards,**

**Joseph J. Bivona III**

**Matthew E. Poynter**

**Reviewer #1:**

This communication provides an open source-based device as a means to study physical exercise, as modelled by wheel-running, in mice. The main issues which are successfully dealt with are both scientific (a device which permits to fix time and/or distance limits according to the experimenter will) and practical (a minor cost, compared to commercially available devices). I encourage the reader to long into the link providing the (detailed) video related to piece assembly.

My sole critics concern bibliographical issues, which however need to be taken into account:
(1) In the 2nd par. of the Introduction, it is indicated that "one drawback of voluntary exercise is the inability to limit running distances". In the authors' mind such a pitfall might probably extend to running times/periods. First, I encourage the authors to read two studies (Dostes et al., Hippocampus 2016; Dubreucq et al., Biol. Psychiatry 2013) wherein the time allowed to run was varied (showing e.g. that exercise-induced neurogenesis is running duration-dependent). Second, whether it is more meaningful to limit running distance or running duration is an open question. Indeed, rodents run for hours but these consist in successive, albeit extremely short, episodes with resting pauses aimed e.g. at drinking, resting, exploring or eating. Moreover, mice do run during the dark phase of the light/dark cycle but their maximal effort is observed during the first 3-4 hours (see the above mentioned study by Dubreucq as an illustration). Taken with the observation that each mouse bears its own active period and its own running speed, it is maybe more relevant to limit the time, rather than the distance, as the variable for wheel locking/unlocking.

**We would like to thank Reviewer #1 for their kind words and constructive response. Our revised submission has included the following references in the second paragraph of the introduction and within the discussion as they further provide the case for appropriate controls and dose dependent effects of exercise:**

**Dostes S, Dubreucq S, Ladevèze E, Marsicano G, Abrous DN, Chaouloff F, et al. Running per se stimulates the dendritic arbor of newborn dentate granule cells in mouse hippocampus in a duration-dependent manner. Hippocampus. 2016;26(3):282-8. doi:** [**https://doi.org/10.1002/hipo.22551**](https://doi.org/10.1002/hipo.22551)**.**

**Dubreucq S, Marsicano G, Chaouloff F. Emotional consequences of wheel running in mice: Which is the appropriate control? Hippocampus. 2011;21(3):239-42. doi:** [**https://doi.org/10.1002/hipo.20778**](https://doi.org/10.1002/hipo.20778)**.**

(2) In the same paragraph, the authors use the word "hyperphysiological" for mouse running behaviour. I sincerely believe that this word should be removed because it might be prone to anthropomorphism. As humans, we consider these distances as enormous. However, mice (and rats) are able to do so without constraint. I encourage the authors to read the excellent review of Sherwin (Animal Behav 1998), which in addition, will allow the authors to change several of their (recent) references by older one which might be more relevant.

**Both Reviewer #1 and #3 have raised concerns about the use of hyperphysiological and upon examination, we agree that this term should be removed from the manuscript. Consequently, we have done so. We have also added the recommended paper (Sherwin, 1998) to the introduction as we enjoyed this perspective on the reasons why animals utilize running wheels. With our background in immunology, we often do not appreciate the behavioral aspects of murine research that the review highlights.**

(3) Legends to the two figures should be separated from the main text and place at the end before the figures

**We have reviewed the PLOS One guidelines and believe the format is correct.**

(4) Discussion:
• The fact that circulating BDNF is not increased by acute exercise is somewhat surprising. How do the authors explain this observation?

**In our experiment, BDNF was not detectable in the serum of both the control and exercise groups. We have removed this sentence from the results and discussion since BDNF and other myokines are not included as data and do not contribute to the manuscript. In humans, BDNF appears to be transiently found after exercise and is returned to baseline after about 10 minutes (Schmidt-Kassow et al 2012). We speculate this rapid utilization may also be the case in mice. It would appear other groups have also had trouble detecting serum BDNF (Delezie et al., 2019, Klein et al., 2010).**

**Schmidt-Kassow M, Schädle S, Otterbein S, Thiel C, Doehring A, Lötsch J, Kaiser J. Kinetics of serum brain-derived neurotrophic factor following low-intensity versus high-intensity exercise in men and women. Neuroreport. 2012 Oct 24;23(15):889-93. doi: 10.1097/WNR.0b013e32835946ca. PMID: 22964969.**

**BDNF is a mediator of glycolytic fiber-type specification in mouse skeletal muscle**

**Julien Delezie, Martin Weihrauch, Geraldine Maier, Rocío Tejero, Daniel J. Ham, Jonathan F. Gill, Bettina Karrer-Cardel, Markus A. Rüegg, Lucía Tabares, Christoph Handschin. Proceedings of the National Academy of Sciences Aug 2019, 116 (32) 16111-16120; DOI: 10.1073/pnas.1900544116**

**Klein AB, Williamson R, Santini MA, Clemmensen C, Ettrup A, Rios M, Knudsen GM, Aznar S. Blood BDNF concentrations reflect brain-tissue BDNF levels across species. Int J Neuropsychopharmacol. 2011 Apr;14(3):347-53. doi: 10.1017/S1461145710000738. Epub 2010 Jul 7. PMID: 20604989.**

• The paragraph comparing forced vs voluntary exercise is incorrect. Asz it stands, it seems that voluntary exercise does not increase corticosterone levels. Besides the observation that it is increased with wheel-running (see relevant figures in ref 22), the fact that corticosterone increases with exercise is logical and is also observed in humans (changing corticosterone to cortisol) because it provides a source of energy.

**Upon reviewing literature, we have included several sources that support the statement that voluntary exercise stimulates reduced levels of corticosterone compared to forced exercise. At Reviewer #1’s suggestion, we have removed the statement that voluntary exercise increases corticosterone levels and replaced it with mention that the wheel itself is an enrichment device.**

• The authors should mention the fact that their device is considered an environmental enrichment which, per se, can alter physiological and behavioural variables (see the abundant bibliography on that subject) hence indicating the need to host control mice with locked wheels (see the abovementioned study by Dostes et al. as an additional illustration).

**We appreciate this suggestion and have added it into the discussion.**

• As mentioned by the authors, "free" wheel-running devices as the one reported in this study allow to easilly measure running performance under voluntary dimensions. However, these devices, by their "free access" dimension, do not permit to selectively measure running motivation. The latter requires operant conditioning paradigms (and hence expensive setups) wherein running access is rendered possible by prior lever pressing (see Iversen, J Exp Anal Behav 1993) or nose poking (see Muguruza et al., JCI Insight 2019) under fixed or variable ratio reinforcement schedules.

**The value of an open-source design is that all files can be modified (and we encourage it). We are in the early stages of creating a resisted LOST-Wheel for hypertrophic exercise. Based on the review by Sherwin and the references suggested by the reviewer, there is certainly a need to incorporate an operant condition feature. We would be willing to collaborate and help design such a device.**

**Reviewer #2:**

The article, regarding the building and validity of the LOSTwheel is very well done. The authors present clear information about materials, assembly, and implementation of the equipment. In addition, they do appropriate tests to verify their observational analysis of the running behavior of the mice. This is true both for not only whether they run or not, as well as for the distance that they ran. Thus, these results are in keeping with many other studies regarding wheel running behavior in mice, indicating that the design of the LOSTwheel is valid for running behavior. Importantly, access to a low cost strategy to make these wheels is invaluable to the many scientists who study this, but do not have large grants to buy the expensive monitoring cages available on the market. Thus, this research has broad implications for impacting the field, including to make this research more widely available. As someone with little experience with electrical builds, I appreciate the very comprehensive manual with clear, step by step pictures and instructions, as well as the online tutorials. The level of detail and clarity provided make this easily replicable.

**We thank Reviewer 2 for their time to review our manuscript and their encouraging comments. One of our goals was to make the assembly and coding of the LOST-Wheel as approachable as possible so that others can implement it in their labs. We hope that this is the case.**

**Reviewer #3:**

This open source, lockable mouse wheel is a useful tool that may enable investigators to conduct mouse exercise trials without limitations due to equipment cost.

Some clarification in the manuscript statements is necessary:
1. Voluntary running wheels that allow restriction of running in time or distance increments are available, although the cost of the equipment may be prohibitive for some laboratories. This is not clear in all sections of the manuscript.

**We have edited a sentence in the introduction to address this point and maintain this perspective through the remainder of the manuscript.**

2. Previously published articles, referenced in the current manuscript (see De Bono et al., 2006) and (Gerecke et al., 2010) illustrating the amount of voluntary running that mice will perform in a given period of time (1 day to 90 days) refute the suggestion that mice will voluntarily run at ‘hyperphysiological” levels.

**We have been convinced through the comments of Reviewer #3 and #1 that the use of hyperphysiological is incorrect and anthropomorphizes the typical distances that mice run. Thus, we have removed these sentences from the manuscript.**

3. Some details about the potential protocols for the LOST-Wheel would be useful if clearly stated in the manuscript. For example, is one week (chronic), the maximum period of time for an exercise protocol (without restarting)?

**We have included additional information into the methods section that indicate the modes that were uploaded.**

How many wheels and COM ports may be run in a single experiment?

**We have added a sentence in the methods to address this question. The number of wheels that can be run is limited by the number of USB ports available on the computer. In our laboratory, we use an inexpensive USB hub splitter to expand the number of wheels connected. The current version of the LOST-Wheel Logger can only accommodate a single wheel. Therefore, to collect data from multiple wheels, the user must open a separate instance of RStudio for each wheel. This is a minor inconvenience but works effectively.**

Approximately how long do the 3D printed wheel and magnet apparatus work efficiently with continued use and cleaning?

**We have added the following paragraph to the discussion to address this comment:**

**Several iterations of the LOST-Wheel were prototyped before using the Hall effect sensor and magnet combination. Originally, the wheel rotated on a rotary encoder, an electro-mechanical part that has a finite number of rotations (30,000-100,000) before wearing out. We also attempted using an infrared sensor, but cage bedding would often block the beam, rendering it useless. The magnet and Hall effect sensor bypass these problems and should remain operational indefinitely. The running surface and shaft can easily be removed and sprayed with ethanol to disinfect between uses. While mice have occasionally chewed the running surface, this has not hindered its balance or performance. We have also designed and provided a guard for the power cord that protects it from destructive animals.**

The Lost-Wheel manual is clear and appears easy to follow to build the wheel and controlling components. The video links provided are very helpful, however, parts of the Assembly video, especially the final assembly portion of the box housing the microcontroller, Nano, and Hall effect sensor, as well as the connection of the components would benefit from a closer view.

**We appreciate the reviewer’s comments on the assembly video and plan to increase the size of the images projected in the sections pertaining to the final assembly during future updates.**

Minor comments:
The use of Gapdh for normalization of the expression of the gene of interest in qPCR is not a stable comparator in all cases (see(Desseille et al., 2017; Xu et al., 2018; Hildyard et al., 2019)). The housekeeping gene 18S rRNA has invariant expression and provides more stable comparison.

**We acknowledge that housekeeping genes are often the topic of debate. Yet, determining the best one may be situation specific. It appears through the references that for dystrophic, atrophic, and recovering muscle, 18S is a proven candidate. For exercise, it may be worth investigating 18S further, since ribosomal biogenesis can be linked to exercise induced hypertrophy.**

**Wang X, Zhao H, Ni J, Pan J, Hua H, Wang Y. Identification of suitable reference genes for gene expression studies in rat skeletal muscle following sciatic nerve crush injury. Mol Med Rep. 2019 May;19(5):4377-4387. doi: 10.3892/mmr.2019.10102. Epub 2019 Mar 28. PMID: 30942461; PMCID: PMC6472138.**

**Regulation of Ribosome Biogenesis in Skeletal Muscle Hypertrophy. Vandré Casagrande Figueiredo and John J. McCarthy. Physiology 2019 34:1, 30-42**

**Journal Requirements:**

When submitting your revision, we need you to address these additional requirements.

1. Please ensure that your manuscript meets PLOS ONE's style requirements, including those for file naming. The PLOS ONE style templates can be found at 
[https://journals.plos.org/plosone/s/file?id=wjVg/PLOSOne_formatting_sample_main_body.pdf](https://nam02.safelinks.protection.outlook.com/?url=https%3A%2F%2Fjournals.plos.org%2Fplosone%2Fs%2Ffile%3Fid%3DwjVg%2FPLOSOne_formatting_sample_main_body.pdf&data=04%7C01%7Cmatthew.poynter%40med.uvm.edu%7C076ee6fc72aa42addbdb08d9ae9c5729%7Ced03ff7aba9f420480a6b226316c919d%7C0%7C0%7C637732809700022532%7CUnknown%7CTWFpbGZsb3d8eyJWIjoiMC4wLjAwMDAiLCJQIjoiV2luMzIiLCJBTiI6Ik1haWwiLCJXVCI6Mn0%3D%7C3000&sdata=D6jmP9tiyo0UIZh0yV54xjenfw6omljbGPOnEvcdAYk%3D&reserved=0) and 
[https://journals.plos.org/plosone/s/file?id=ba62/PLOSOne_formatting_sample_title_authors_affiliations.pdf](https://nam02.safelinks.protection.outlook.com/?url=https%3A%2F%2Fjournals.plos.org%2Fplosone%2Fs%2Ffile%3Fid%3Dba62%2FPLOSOne_formatting_sample_title_authors_affiliations.pdf&data=04%7C01%7Cmatthew.poynter%40med.uvm.edu%7C076ee6fc72aa42addbdb08d9ae9c5729%7Ced03ff7aba9f420480a6b226316c919d%7C0%7C0%7C637732809700032487%7CUnknown%7CTWFpbGZsb3d8eyJWIjoiMC4wLjAwMDAiLCJQIjoiV2luMzIiLCJBTiI6Ik1haWwiLCJXVCI6Mn0%3D%7C3000&sdata=Au8lqnKDoDHwHHmmqjQVxzOJxF35DR5OpPzBXufEIMI%3D&reserved=0)

**We have modified our revised manuscript to comply with PLOS ONE’s style requirements.**

2. We note that the grant information you provided in the ‘Funding Information’ and ‘Financial Disclosure’ sections do not match. When you resubmit, please ensure that you provide the correct grant numbers for the awards you received for your study in the ‘Funding Information’ section

**We have confirmed our grant numbers on the manuscript to match those on the Editorial Manager portal.**

3. We note that you have included the phrase “data not shown” in your manuscript. Unfortunately, this does not meet our data sharing requirements. PLOS does not permit references to inaccessible data. We require that authors provide all relevant data within the paper, Supporting Information files, or in an acceptable, public repository. Please add a citation to support this phrase or upload the data that corresponds with these findings to a stable repository (such as Figshare or Dryad) and provide and URLs, DOIs, or accession numbers that may be used to access these data. Or, if the data are not a core part of the research being presented in your study, we ask that you remove the phrase that refers to these data.

**We have determined that the data omitted do not add value to the manuscript and have removed the sentences.**

4. Please include your full ethics statement in the ‘Methods’ section of your manuscript file. In your statement, please include the full name of the IRB or ethics committee who approved or waived your study, as well as whether or not you obtained informed written or verbal consent. If consent was waived for your study, please include this information in your statement as well.

**We have updated our methods to include the UVM IACUC protocol number.**

5. Please review your reference list to ensure that it is complete and correct. If you have cited papers that have been retracted, please include the rationale for doing so in the manuscript text, or remove these references and replace them with relevant current references. Any changes to the reference list should be mentioned in the rebuttal letter that accompanies your revised manuscript. If you need to cite a retracted article, indicate the article’s retracted status in the References list and also include a citation and full reference for the retraction notice.

**We have searched our references with the retraction watch database and found no overlap.**
